# A Combination of Celecoxib and Glucosamine Sulfate Has Anti-Inflammatory and Chondroprotective Effects: Results from an In Vitro Study on Human Osteoarthritic Chondrocytes

**DOI:** 10.3390/ijms22168980

**Published:** 2021-08-20

**Authors:** Sara Cheleschi, Sara Tenti, Stefano Giannotti, Nicola Veronese, Jean-Yves Reginster, Antonella Fioravanti

**Affiliations:** 1 Rheumatology Unit, Department of Medicine, Surgery and Neuroscience, Azienda Ospedaliera Universitaria Senese, Policlinico Le Scotte, 53100 Siena, Italy; saracheleschi@hotmail.com (S.C.); sara_tenti@hotmail.it (S.T.); fioravanti7@virgilio.it (A.F.); 2 Section of Orthopedics and Traumatology, Department of Medicine, Surgery and Neurosciences, University of Siena, Policlinico Le Scotte, 53100 Siena, Italy; stefano.giannotti@unisi.it; 3 Geriatric Unit, Department of Internal Medicine and Geriatrics, University of Palermo, Viale Scaduto, 90100 Palermo, Italy; 4 Department of Public Health, Epidemiology and Health Economics, University of Liège, Quartier Hôpital, Avenue Hippocrate 13, Bât. B23, 4000 Liège, Belgium; jyreginster@uliege.be

**Keywords:** celecoxib, glucosamine sulfate, osteoarthritis, chondrocytes, inflammation, chondroprotection, oxidative stress, NF-κB

## Abstract

This study investigated the possible anti-inflammatory and chondroprotective effects of a combination of celecoxib and prescription-grade glucosamine sulfate (GS) in human osteoarthritic (OA) chondrocytes and their possible mechanism of action. Chondrocytes were treated with celecoxib (1.85 µM) and GS (9 µM), alone or in combination with *IL-1β* (10 ng/mL) and a specific nuclear factor (NF)-κB inhibitor (BAY-11-7082, 1 µM). Gene expression and release of some pro-inflammatory mediators, metalloproteinases (*MMPs*), and type II collagen (Col2a1) were evaluated by qRT-PCR and ELISA; apoptosis and mitochondrial superoxide anion production were assessed by cytometry; B-cell lymphoma (BCL)2, antioxidant enzymes, and *p50* and *p65* NF-κB subunits were analyzed by qRT-PCR. Celecoxib and GS alone or co-incubated with *IL-1β* significantly reduced expression and release of cyclooxygenase (*COX)-2*, prostaglandin (*PG)E2*, *IL-1β*, *IL-6*, tumor necrosis factor (*TNF)-α*, and *MMPs*, while it increased *Col2a1*, compared to baseline or *IL-1β*. Both drugs reduced apoptosis and superoxide production; reduced the expression of superoxide dismutase, catalase, and nuclear factor erythroid; increased *BCL2*; and limited *p50* and *p65*. Celecoxib and GS combination demonstrated an increased inhibitory effect on *IL-1β* than that observed by each single treatment. Drugs effects were potentiated by pre-incubation with BAY-11-7082. Our results demonstrated the synergistic effect of celecoxib and GS on OA chondrocyte metabolism, apoptosis, and oxidative stress through the modulation of the NF-κB pathway, supporting their combined use for the treatment of OA.

## 1. Introduction

Osteoarthritis (OA) is the most common degenerative musculoskeletal disorder that affects the entire joint. Its main symptoms are chronic pain, functional limitation, instability, and deformity, with a considerable reduction in quality of life; therefore, OA is considered the leading cause of disability and impairment in middle-aged and older people worldwide and represents a real burden to health care systems [1,2]. The pathogenesis of OA is complex and remains largely unknown; however, it is assumed that multiple factors including aging, gender, joint injury, obesity, and metabolic factors contribute to the onset and the progression of the disease [1]. Furthermore, the risk of developing OA is increased by physical inactivity and by a low-fiber diet rich in sugar and saturated fats. All these different factors are associated with an alteration of composition and function of the gut microbiota, causative of low-grade inflammation, which is an important contributor to joint damage in OA [3,4]. This microbial dysbiosis could represent the missing link between the different conditions contributing to OA pathogenesis, suggesting microbiota as a new pharmacological strategy for OA management [5,6].

Current strategies for the management of OA include a combination of pharmacological and/or non-pharmacological approaches. Among the pharmacological treatments, the updated European Society for Clinical and Economic Aspects of Osteoporosis and Osteoarthritis (ESCEO) algorithm recommends chronic symptomatic slow-acting drugs for OA (SYSADOAs), such as prescription-grade glucosamine sulfate (GS) or chondroitin sulfate (CS), as first-line long-term background treatment, and as-needed paracetamol as a short-term step to rescue analgesia only [7]. Topical nonsteroidal anti-inflammatory drugs (NSAIDs) may be added to the treatment regimen in step 1 if the patient is still symptomatic after establishing appropriate background pharmacological therapy with SYSADOAs, and rescue analgesia with paracetamol provides insufficient symptom relief. The use of oral NSAIDs (selective or non-selective) is proposed as second step, and short-term therapy, with selective COX-2 inhibitors (COXIBs) preferred in the case of increased gastro-intestinal risk [7]. Despite this protocol being endorsed by several groups of experts from around the world, the recommendation to use SYSADOAs as first-line background treatment for knee OA is not endorsed by other respected scientific societies [8,9,10,11,12]. Recently, the ESCEO working group reinforced the use of GS and CS as first-line long-term treatment for their activity on gut microbiota. Indeed, they have limited intestinal absorption and are predominantly utilized as substrates by the gut microbiota. They may have prebiotic properties and exert their therapeutic effects through gut bacterial pathways [5,13,14].

Traditionally, COXIBs have been widely used for their well-established analgesic and anti-inflammatory properties for the treatment of OA; in recent years, growing evidence raised the question of whether COXIBs can be viewed as disease-modifying OA drugs (DMOADs), able to reduce cartilage degradation and slow down OA disease progression [15,16,17,18]. Different in vivo and in vitro studies focused on the potential role of celecoxib as DMOADs [15,19,20,21,22]. In particular, this drug showed the ability to decrease the production of prostaglandin *(PG)E2*, interleukin *(IL)-1β*, tumor necrosis factor *(TNF)-α*, and nitric oxide (NO) release, and it increased the synthesis of proteoglycans and type II collagen (Col2a1) in human OA cartilage and chondrocytes [21,22,23]. Furthermore, celecoxib reduced the synthesis of metalloproteinases (*MMPs*), apoptosis, and the activation of nuclear factor (NF)-κB and receptor activator of NF-κB ligand (RANKL) in OA chondrocytes, fibroblast-like synoviocytes, and subchondral bone osteoblasts [21,24,25].

GS is an amino-monosaccharide and a natural constituent of long-chain glycosaminoglycans present in human tissues, with the highest part in cartilage matrix. The high-quality prescription-grade crystalline GS formulation is widely used for the treatment of OA due to the demonstrated symptomatic effects as well as disease-modifying properties [26,27,28,29,30,31]. Furthermore, its specific role in cartilage and chondrocyte metabolism has been also demonstrated [30,32]. Indeed, different studies showed the effects of GS in reducing expression of *COX-2*, PGE2 production, and inhibiting activation of the NF-κB pathway in human OA chondrocytes and synoviocytes [33,34,35]. Furthermore, GS promoted chondrocyte proliferation and proteoglycan production, while it decreased the expression of inducible form of nitric oxide (*iNOS*), *IL-6*, and *TNF-α* and matrix degrading factors [35,36,37,38].

The aim of the present study was to investigate the possible anti-inflammatory and chondroprotective effects of celecoxib and GS, tested alone or in combination, in human OA chondrocytes in the presence of *IL-1β*. In particular, matrix-degrading enzymes (*MMP-1*, *MMP-3*, *MMP-13*), Col2a1, and cytokines (*COX-2*, *PGE2*, *IL-1β*, *IL-6*, *TNF-α*) were analyzed at their expression levels as well as at their release in the supernatant. Cell viability, the ratio of apoptosis, and the mRNA levels of the anti-apoptotic mediator B-cell lymphoma 2 (BCL)2 were also assessed. Furthermore, the production of mitochondrial superoxide anion and the gene levels of the main antioxidant enzymes—superoxide dismutase *(SOD)-2*, catalase (*CAT*), glutathione peroxidase (GPx)4, and nuclear factor erythroid 2 like 2 (*NRF2*)—were evaluated. Finally, possible regulation of the NF-κB pathway was detected.

## 2. Results

### 2.1. Celecoxib and GS Attenuate Inflammation

**Figure 1:**
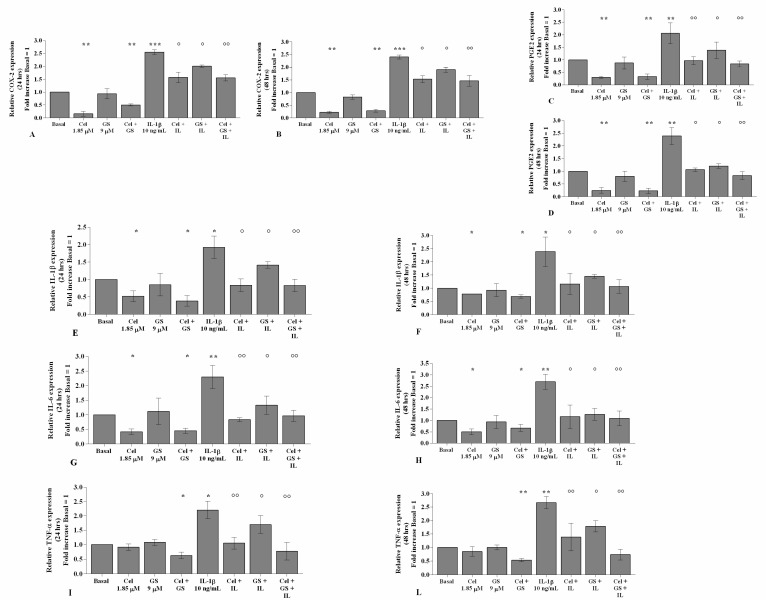
Celecoxib and GS reduce the expression of pro-inflammatory cytokines. Human osteoarthritic (OA) chondrocytes were incubated for 24 and 48 h with celecoxib (1.85 µM) and prescription-grade glucosamine sulfate (GS) (9 µM) (2 h of pre-treatment) in the presence of interleukin *(IL)-1β* (10 ng/mL). (**A**–**L**) Expression levels of cyclooxygenase (*COX)-2*, prostaglandin (*PG)E2*, *IL-1β*, *IL-6*, and tumor necrosis factor *(TNF)-α* analyzed by quantitative real-time PCR. The gene expression was referenced to the ratio of the value of interest and the value of the basal condition, reported equal to 1. Data were represented as mean ± SD of triplicate values. * *p* < 0.05, ** *p* < 0.01, *** *p* < 0.001 versus basal condition. ° *p* < 0.05, °° *p* < 0.01 versus *IL-1β*.

**Figure 2:**
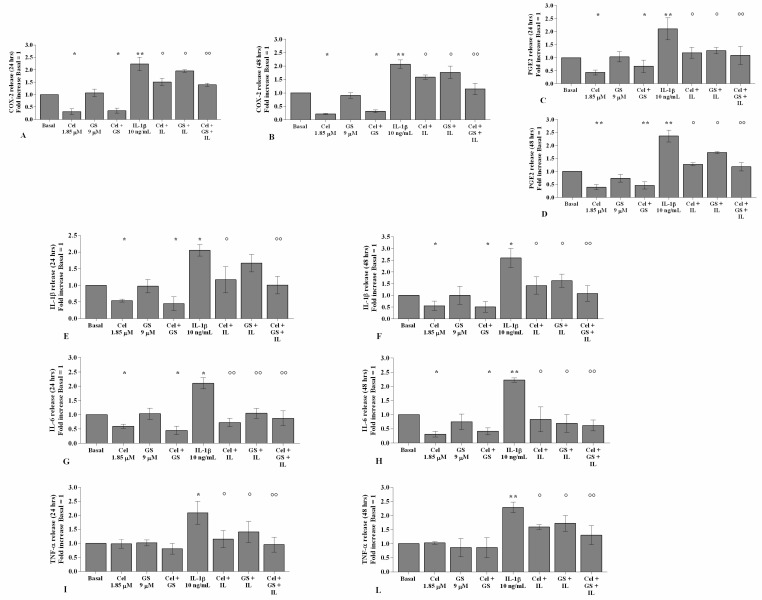
Celecoxib and GS reduce the release of pro-inflammatory cytokines. Human osteoarthritic (OA) chondrocytes were incubated for 24 and 48 h with celecoxib (1.85 µM) and prescription-grade glucosamine sulfate (GS) (9 µM) (2 h of pre-treatment) in the presence of interleukin *(IL)-1β* (10 ng/mL). (**A**–**L**) Total amount of cyclooxygenase *(COX)-2*, prostaglandin *(PG)E2*, *IL-1β*, *IL-6*, and tumor necrosis factor *(TNF)-α*, released in the supernatant, analyzed by ELISA assay. The total amount was referenced to the ratio of the value of interest and the value of the basal condition, reported equal to 1. Data were represented as mean ± SD of triplicate values. * *p* < 0.05, ** *p* < 0.01 versus basal condition. ° *p* < 0.05, °° *p* < 0.01 versus *IL-1β*.

Figure 1 and Figure 2 show the effects of treatment with celecoxib (1.85 µM) and GS (9 µM), for 24 h and 48 h, on gene expression and supernatant release of the main pro-inflammatory mediators, in OA chondrocytes stimulated or not with *IL-1β*.

The treatment of OA chondrocytes with celecoxib, tested alone or in combination with GS, significantly reduced *COX-2*, *PGE2*, *IL-1β*, and *IL-6* gene expression and release in comparison to basal conditions (*p* < 0.05, *p* < 0.01), while no changes in TNF-α were observed (Figure 1A–L, Figure 2A–L). The incubation with GS alone did not show any detectable modification compared to baseline (Figure 1A–L, Figure 2A–L).

Stimulation of the cells with *IL-1β* caused a significant up-regulation of all analyzed genes (*p* < 0.05, *p* < 0.01). Pre-treatment of the cells with celecoxib or GS significantly limited the negative effect of *IL-1β*, in particular when the drugs were tested in combination, both at 24 and 48 h (*p* < 0.05, *p* < 0.01) (Figure 1A–L, Figure 2A–L).

### 2.2. Effects of Celecoxib and GS on Cellular Survival and Apoptosis

**Figure 3:**
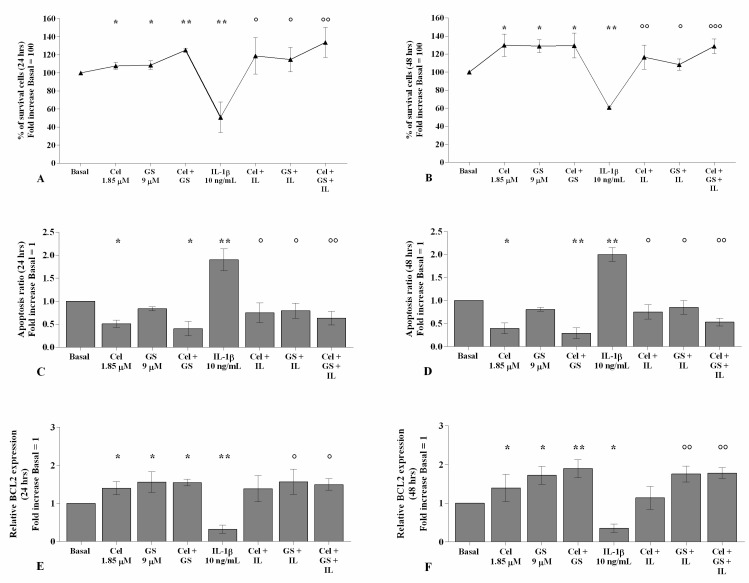
Celecoxib and GS regulate viability and apoptosis. Human osteoarthritic (OA) chondrocytes were incubated for 24 and 48 h with celecoxib (1.85 µM) and prescription-grade glucosamine sulfate (GS) (9 µM) (2 h of pre-treatment) in the presence of interleukin *(IL)-1β* (10 ng/mL). (**A**,**B**) Evaluation of cell viability by MTT assay. (**C**,**D**) Apoptosis detection performed by flow cytometry analysis and measured with Annexin Alexa fluor 488 assay. Data were expressed as the percentage of positive cells for Annexin-V and propidium iodide (PI) staining. (**E**,**F**) Expression levels of B-cell lymphoma (BCL2) analyzed by quantitative real-time PCR. The percentage of surviving cells, the ratio of apoptosis, and the gene expression were referenced to the ratio of the value of interest and the value of the basal condition, reported equal to 100 or 1. Data were represented as mean ± SD of triplicate values. * *p* < 0.05, ** *p* < 0.01 versus basal condition. ° *p* < 0.05, °° *p* < 0.01, °°° *p* < 0.001 versus *IL-1β*.

The incubation of chondrocytes with celecoxib or GS alone significantly increased the percentage of survival cells, reduced the apoptotic rate, and up-regulated the gene expression of the anti-apoptotic marker *BCL2* (*p* < 0.05), in comparison to basal conditions, at both analyzed time points (Figures S1 and S2, Figure 3A–F); this trend was maintained and enhanced when the compounds were tested in combination (*p* < 0.05, *p* < 0.01). On the contrary, the stimulus with *IL-1β* significantly reduced viability (*p* < 0.01) and induced apoptosis (*p* < 0.01), which were counteracted by the pre-incubation of the cells with celecoxib and GS alone and, especially, in combination (*p* < 0.05, *p* < 0.01) (Figure 3A–F).

### 2.3. Celecoxib and GS Modulate the Oxidant/Antioxidant System

**Figure 4:**
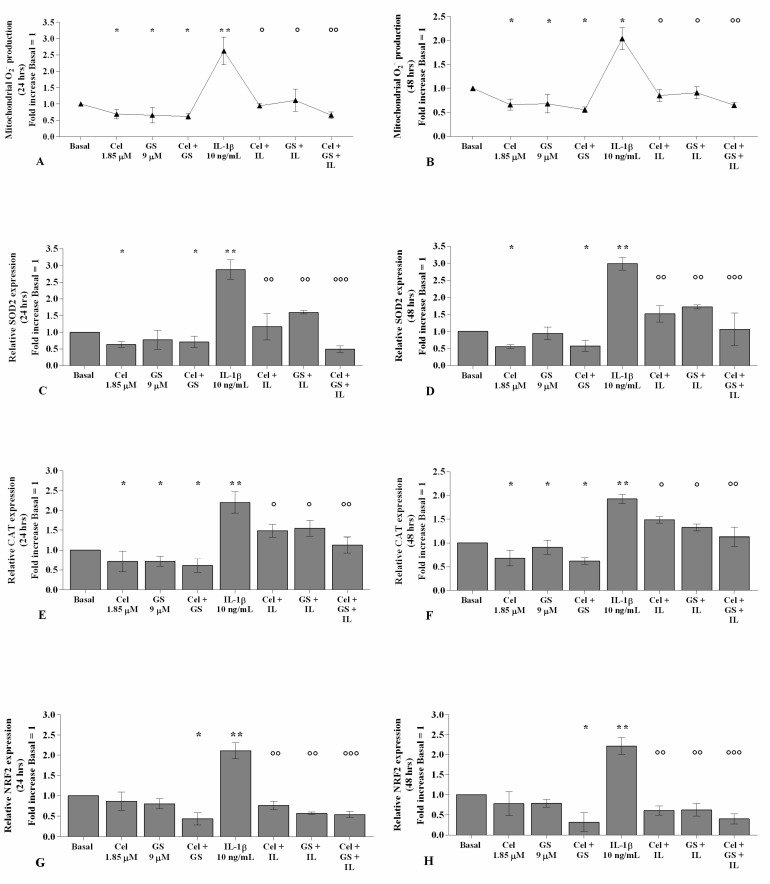
Celecoxib and GS regulate oxidative stress balance. Human osteoarthritic (OA) chondrocytes were incubated for 24 and 48 h with celecoxib (1.85 µM) and prescription-grade glucosamine sulfate (GS) (9 µM) (2 h of pre-treatment) in the presence of interleukin *(IL)-1β* (10 ng/mL). (**A**,**B**) Mitochondrial superoxide anion production evaluated by MitoSox Red staining at flow cytometry. (**C**–**H**) Expression levels of superoxide dismutase *(SOD)-2*, catalase (*CAT*), and nuclear factor erythroid 2 like 2 (*NRF2*) analyzed by quantitative real-time PCR. The production of superoxide anion and the gene expression were referenced to the ratio of the value of interest and the value of basal condition, reported equal to 1. Data were represented as mean ± SD of triplicate values. * *p* < 0.05, ** *p* < 0.01 versus basal condition. ° *p* < 0.05, °° *p* < 0.01, °°° *p* < 0.001 versus *IL-1β*.

The potential role of celecoxib and GS in the regulation of oxidant/antioxidant balance in chondrocytes stimulated with *IL-1β* was reported in Figure S3 and Figure 4. Celecoxib and GS, analyzed alone or in combination, significantly reduced the production of mitochondrial superoxide anion and the gene expression of *SOD-2*, *CAT*, and *NRF2*, at 24 and 48 h, compared to baseline (*p* < 0.05, Figure 4A–H). Conversely, *IL-1β* stimulus induced mitochondrial ROS production and mRNA levels of the antioxidant enzymes (*p* < 0.05, *p* < 0.01); otherwise, pre-treatment with either celecoxib or GS limited the negative effect of *IL-1β* on redox balance, with an enhancement when the drugs were used in combination, both at 24 and 48 h (*p* < 0.01, *p* < 0.001) (Figure 4A–H).

### 2.4. Celecoxib and GS Regulate Cartilage Turnover

**Figure 5:**
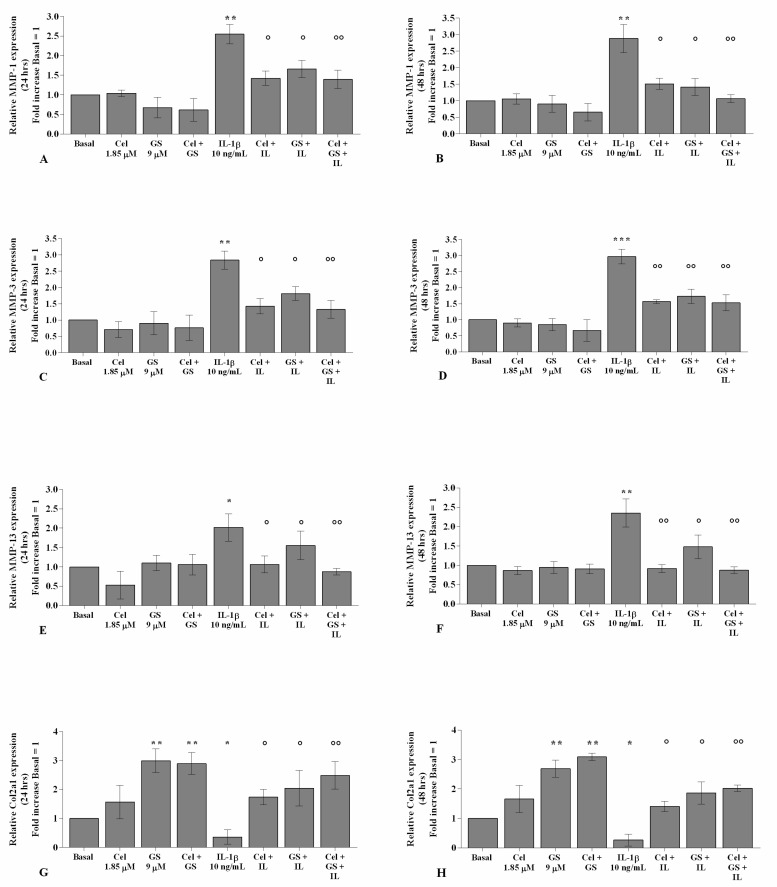
Celecoxib and GS regulate cartilage metabolism. Human osteoarthritic (OA) chondrocytes were incubated for 24 and 48 h with celecoxib (1.85 µM) and prescription-grade glucosamine sulfate (GS) (9 µM) (2 h of pre-treatment) in the presence of interleukin *(IL)-1β* (10 ng/mL). (**A**–**H**) Expression levels of metalloproteinase *(MMP)-1, -3, -13*, and type II collagen (*Col2a1*), analyzed by quantitative real-time PCR. The gene expression was referenced to the ratio of the value of interest and the value of basal condition, reported equal to 1. Data were represented as mean ± SD of triplicate values. * *p* < 0.05, ** *p* < 0.01, *** *p* < 0.001 versus basal condition. ° *p* < 0.05, °° *p* < 0.01 versus *IL-1β*.

**Figure 6:**
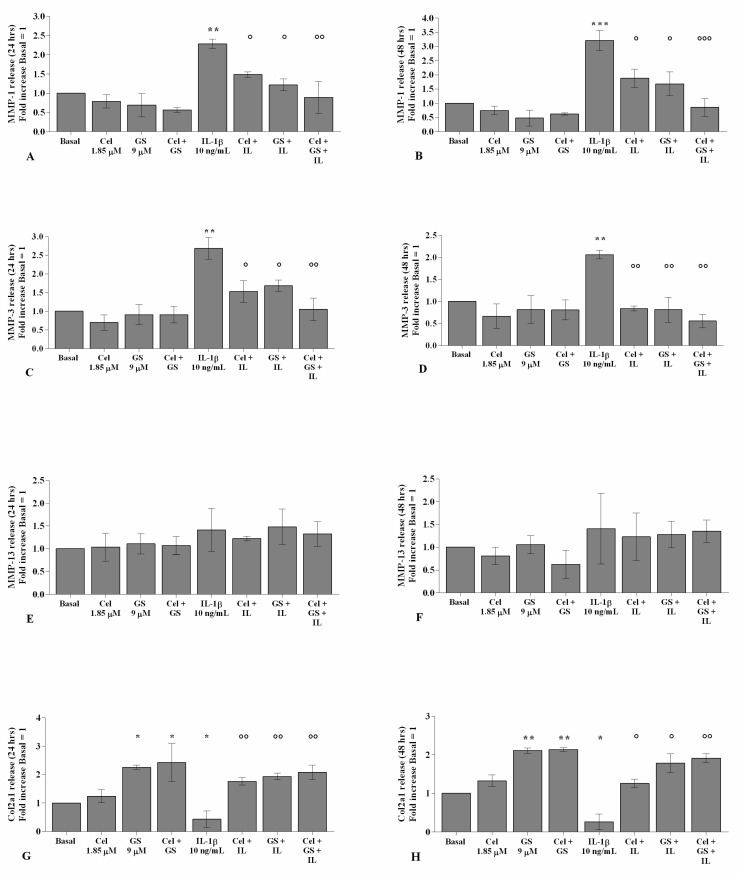
Celecoxib and GS regulate cartilage metabolism. Human osteoarthritic (OA) chondrocytes were incubated for 24 and 48 h with celecoxib (1.85 µM) and prescription-grade glucosamine sulfate (GS) (9 µM) (2 h of pre-treatment) in the presence of interleukin *(IL)-1β* (10 ng/mL). (**A**–**H**) Total amount of metalloproteinase *(MMP)-1*, *-3*, *-13*, and type II collagen (Col2a1) released in the supernatant, analyzed by ELISA assay. The total amount was referenced to the ratio of the value of interest and the value of the basal condition, reported equal to 1. Data were represented as mean ± SD of triplicate values. * *p* < 0.05, ** *p* < 0.01, *** *p* < 0.001 versus basal condition. ° *p* < 0.05, °° *p* < 0.01, °°° *p* < 0.001 versus *IL-1β*.

As reported in Figure 5 and Figure 6, the gene expression and supernatant release of the matrix-degrading enzymes—*MMP-1*, *MMP-3,* and *MMP-13*—did not show any significant change in OA chondrocytes incubated, for 24 and 48 h, with celecoxib or GS alone in comparison to basal conditions (Figure 5A–F, Figure 6A–F). GS significantly increased the expression and release of *Col2a1* (*p* < 0.05, *p* < 0.01) when tested alone or in combination with celecoxib (Figure 5G–H, Figure 6G–H).

The statistically significant increase in *MMP-1*, *MMP-3*, and *MMP-13* and the down-regulation of *Col2a1* induced by *IL-1β* stimulus (*p* < 0.05, *p* < 0.01, *p* < 0.001) were partially inhibited by pre-treatment of the cells with celecoxib or GS. Co-incubation of OA chondrocytes with both drugs induced a more significant exacerbation by the combination of them, both at 24 and 48 h (*p* < 0.05, *p* < 0.01, *p* < 0.001) (Figure 5A–H, Figure 6A–H).

### 2.5. Celecoxib and GS Reduce NF-κB Signaling Pathway Activation

**Figure 7:**
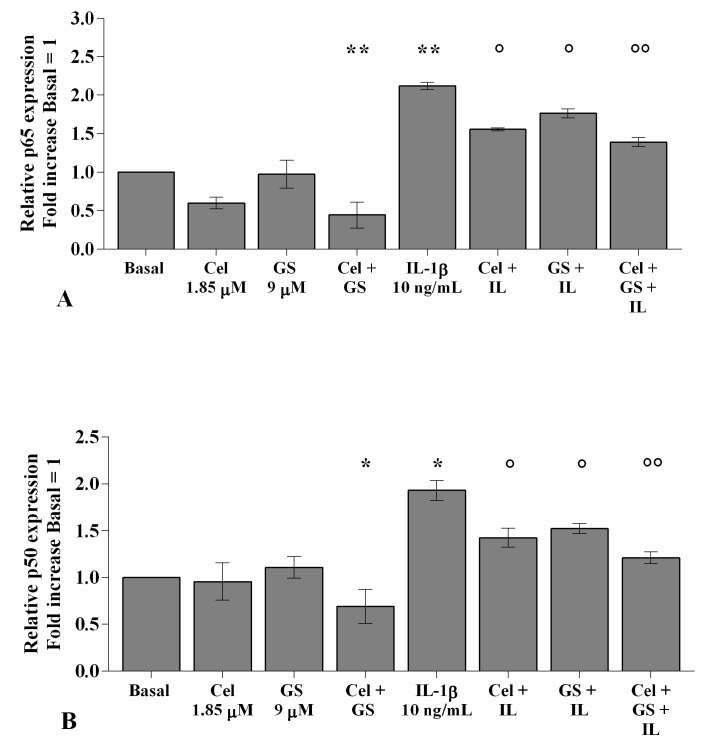
Celecoxib and GS modulate the NF-κB signaling pathway. Human osteoarthritic (OA) chondrocytes were incubated for 4 h with celecoxib (1.85 µM) and prescription-grade glucosamine sulfate (GS) (9 µM) (2 h of pre-treatment) in the presence of interleukin *(IL)-1β* (10 ng/mL). (**A**,**B**) Expression levels of *p65* and *p50* subunits were analyzed by quantitative real-time PCR. The gene expression was referenced to the ratio of the value of interest and the value of basal condition, reported equal to 1. Data were represented as mean ± SD of triplicate values. * *p* < 0.05, ** *p* < 0.01 versus basal condition. ° *p* < 0.05, °° *p* < 0.01 versus *IL-1β*.

Figure 7 shows the effects of celecoxib and GS on NF-κB signaling pathway regulation. PCR real-time analysis reported no significant changes in OA chondrocytes incubated for 4 h with celecoxib or GS alone in comparison to baseline, except for the combination of them, which induced a significant reduction in *p65* and *p50* subunits gene expression (*p* < 0.01, *p* < 0.05, Figure 7A,B). As expected, the significant up-regulation of *p65* and *p50* gene expression induced by *IL-1β* stimulus (*p* < 0.01, *p* < 0.05) was partially counteracted by the pre-treatment of the cells with celecoxib or GS (*p* < 0.05) and, especially, when the drugs were tested in combination (*p* < 0.01) (Figure 7A,B).

### 2.6. NF-κB Inhibitor Enhances Celecoxib and GS-Induced Effects

**Figure 8:**
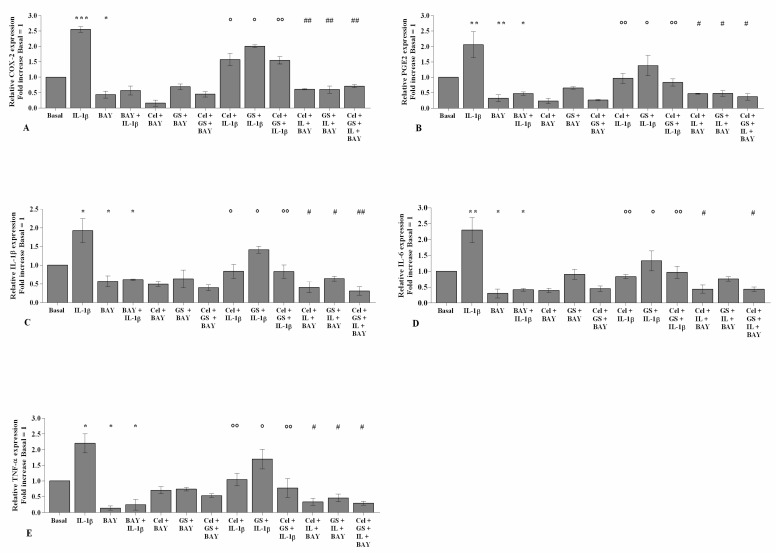
BAY 11-7082 influences celecoxib and GS effects on pro-inflammatory cytokines. Human osteoarthritic (OA) chondrocytes were incubated for 24 h with celecoxib (1.85 µM) and prescription-grade glucosamine sulfate (GS) (9 µM) (2 h of pre-treatment) in the presence of interleukin *(IL)-1β* (10 ng/mL) and BAY 11-7082 1 μM (NF-κB inhibitor, 2 h of pre-treatment). (**A**–**E**) Expression levels of cyclooxygenase (*COX)-2*, prostaglandin *(PG)E2*, *IL-1β*, *IL-6*, and tumor necrosis factor *(TNF)-α* analyzed by quantitative real-time PCR. The gene expression was referenced to the ratio of the value of interest and the value of basal condition, reported equal to 1. Data were represented as mean ± SD of triplicate values. * *p* < 0.05, ** *p* < 0.01, *** *p* < 0.001 versus basal condition. ° *p* < 0.05, °° *p* < 0.01 versus *IL-1β*. # *p* < 0.05, ## *p* < 0.01 versus celecoxib or GS plus BAY.

**Figure 9:**
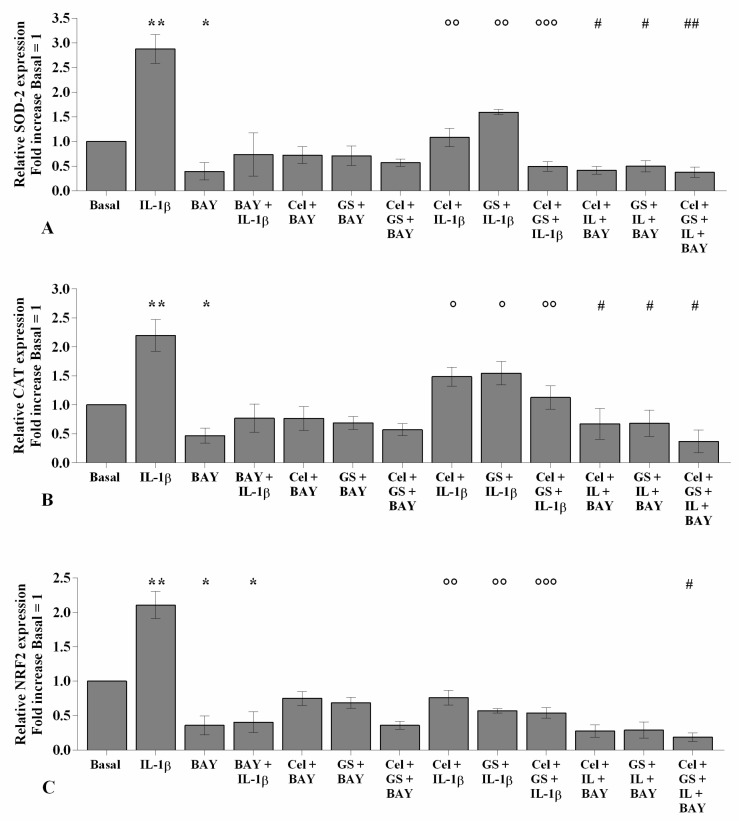
BAY 11-7082 influences celecoxib and GS effects on anti-oxidant enzymes expression. Human osteoarthritic (OA) chondrocytes were incubated for 24 h with celecoxib (1.85 µM) and prescription-grade glucosamine sulfate (GS) (9 µM) (2 h of pre-treatment) in the presence of interleukin *(IL)-1β* (10 ng/mL) and BAY 11-7082 1 μM (NF-κB inhibitor, 2 h of pre-treatment). (**A**–**C**) Expression levels of superoxide dismutase *(SOD)-2*, catalase (*CAT*), and nuclear factor erythroid 2 like 2 (*NRF2*) analyzed by quantitative real-time PCR. The gene expression was referenced to the ratio of the value of interest and the value of basal condition, reported equal to 1. Data were represented as mean ± SD of triplicate values. * *p* < 0.05, ** *p* < 0.01 versus basal condition. ° *p* < 0.05, °° *p* < 0.01, °°° *p* < 0.001 versus *IL-1β*. # *p* < 0.05, ## *p* < 0.01 versus celecoxib or GS plus BAY.

**Figure 10:**
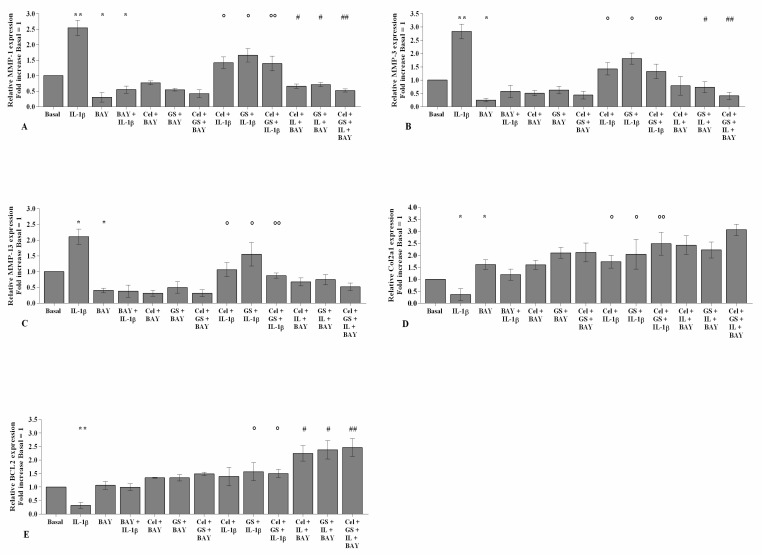
BAY 11-7082 enhances celecoxib and GS effects on cartilage metabolism. Human osteoarthritic (OA) chondrocytes were incubated for 24 h with celecoxib (1.85 µM) and prescription-grade glucosamine sulfate (GS) (9 µM) (2 h of pre-treatment) in the presence of interleukin *(IL)-1β* (10 ng/mL) and BAY 11-7082 1 μM (NF-κB inhibitor, 2 h of pre-treatment). (**A**–**E**) Expression levels of metalloproteinase (*MMP)-1*, *-3*, *-13*, type II collagen (*Col2a1*), and B-cell lymphoma (*BCL2*) analyzed by quantitative real-time PCR. The gene expression was referenced to the ratio of the value of interest and the value of basal condition, reported equal to 1. Data were represented as mean ± SD of triplicate values. * *p* < 0.05, ** *p* < 0.01 versus basal condition. ° *p* < 0.05, °° *p* < 0.01 versus *IL-1β*. # *p* < 0.05, ## *p* < 0.01 versus celecoxib or GS plus BAY.

To demonstrate the involvement of the NF-κB signaling pathway in mediating celecoxib and GS-induced effects on inflammatory, apoptotic, and oxidative stress mediators, OA chondrocytes were pre-incubated with a specific NF-κB inhibitor (BAY 11-7082, IKKα/β) (Figure 8, Figure 9 and Figure 10).

The transcriptional levels of *COX-2*, *PGE2*, *IL-1β*, *IL-6*, *TNF-α* (Figure 8), *SOD-2*, *CAT*, *NRF2* (Figure 9), *MMP-1*, *MMP-3*, and *MMP-13* (Figure 10) were significantly reduced (*p* < 0.01, *p* < 0.001) in OA cells incubated with BAY 11-7082, while an up-regulation of *Col2a1* mRNA levels was observed (*p* < 0.05, Figure 10) in comparison to the basal condition and *IL-1β*.

The co-treatment of the cells with BAY 11-7082 and celecoxib or GS, alone or in combination, did not show any significant difference in target gene expression with respect to what was observed in OA chondrocytes incubated with BAY 11-7082 alone (Figure 8, Figure 9 and Figure 10).

Furthermore, incubation of the NF-κB inhibitor with celecoxib and GS, in the presence of *IL-1β* stimulus, significantly reduced the expression levels of the analyzed genes beyond that caused by each treatment and, in particular, limited that induced by *IL-1β* (Figure 8, Figure 9 and Figure 10).

## 3. Discussion

The goal of the present research was to examine the possible anti-inflammatory and chondroprotective effects of a combination of celecoxib and GS on inflammation, cartilage turnover, apoptosis, and oxidative stress in human OA chondrocyte cultures, and the potential mechanism of action underlying their favorable effects.

The concentrations of celecoxib and GS tested in the present study seem to be the most appropriate to reflect the mean plasma concentration of drugs reaching the systemic circulation, and they were chosen according to those used by other authors [39,40]. Furthermore, our chondrocytes were grown in culture medium containing low concentrations of glucose (DMEM with 25 mmol/L of glucose), to avoid any possible competition with GS for glucose transporters, thus impeding adequate GS uptake into the cells [35,41]. Finally, the cultures were stimulated with *IL-1β*, a potent pro-inflammatory cytokine generally used in in vitro models to mimic the circumstances driving to in vivo cartilage degradation and inflammatory status [35,42].

Our first result confirmed the significant increase in gene expression and supernatant release of the main pro-inflammatory cytokines, *IL-1β*, *IL-6*, *TNF-α*, and *PGE2*, the latter probably related to the increase in *COX-2*, in OA chondrocytes stimulated with *IL-1β*, as previously reported [22,35]. The incubation of the cells with celecoxib or GS counteracted the negative effects of *IL-1β* on these mediators, similarly to what was observed on OA chondrocytes and OA canine models by other authors [19,22,23,33,35]. Furthermore, we demonstrated, for the first time, a synergistic anti-inflammatory effect of celecoxib and GS, when tested in combination, in *IL-1β*-treated chondrocytes, with an activity retention until 48 h of treatment.

The regulation of chondrocytes survival is important for the maintenance of a proper cartilage structure and function. Indeed, morphological changes due to an increase in apoptosis are typical features of OA damage [43]. The analysis of viability and apoptosis carried out in the present study showed a reduction in survival and an increase in apoptotic chondrocytes in the presence of *IL-1β*, which appeared in agreement with previous research [22,44,45]. On the contrary, celecoxib and GS, tested alone or in combination, increased viability and decreased apoptosis, with a concomitant up-regulation of the anti-apoptotic marker *BCL2*; the anti-apoptotic effect of our drugs was also demonstrated in OA cells stimulated with *IL-1β*. These results are consistent with other authors who reported the ability of both compounds to promote proliferation and reduce the apoptosis rate of OA chondrocytes with or without the influence of the negative stimulus of *IL-1β* [21,22,38,46]. Furthermore, we firstly observed that the anti-apoptotic activity of celecoxib and GS was increased when *IL-1β*-stimulated chondrocytes were simultaneously incubated with the drugs, at both 24 and 48 h of treatment.

Accumulating evidence indicates ROS and reactive nitrogen species as important mediators of cartilage damage that occurs in OA; the failure in oxidant/antioxidant balance in OA chondrocytes induces an altered redox status, which promotes cartilage breakdown and makes cells more susceptible to oxidant-mediated apoptosis [2]. In the current study, the assessment of oxidative stress showed an increase in mitochondrial superoxide anion production and antioxidant enzymes expression, *SOD2*, *CAT*, and of the transcriptional factor *NRF2*, in OA chondrocytes exposed to *IL-1β*, in agreement with our previous findings [22]. Furthermore, we demonstrated the ability of celecoxib or GS to decrease ROS release and antioxidant enzyme gene levels, and their effect was also maintained following *IL-1β* stimulus, in line with the current literature. Indeed, some authors reported the reduction of superoxide anion production and SOD2 activity, caused by celecoxib, in OA chondrocytes stimulated with *IL-1β* [22,47]. Similarly, a decrease in superoxide anion and inducible nitric oxide synthase (iNOS) expression, induced by *IL-1β*, was found in human OA chondrocytes treated with GS [48,49,50]. Interestingly, our results further demonstrated that the activation of oxidative stress caused by *IL-1β* was strongly reversed by the simultaneous treatment of the cells with celecoxib and GS, indicating a more efficacious anti-oxidant effect of the studied drugs when used in combination.

The activation of different matrix-degrading enzymes, such as *MMP-1*, *MMP-3*, and *MMP-13*, and the consequent degradation of proteoglycans and Col2a1 represents one of main characteristics of OA and has been amply demonstrated in human OA articular cartilage and chondrocytes following *IL-1β* stimulus [9,17,18,41,45,46]. In agreement with these data, in the present paper, we observed a significant increase in *MMP-1*, *-3*, and *-13* and a reduction in *Col2a1* gene expression and supernatant release in *IL-1β*-stimulated OA chondrocytes. The incubation of our cultures with celecoxib or GS alone did not modify the studied *MMPs* and Col2a1, according to other findings [47,48], while it was able to counteract the negative effect of *IL-1β* on these factors, especially when the drugs were co-incubated, until 48 h of treatment, suggesting a possible anti-catabolic effect of the tested compounds. In a similar manner, other authors reported a reduction in *MMP-1*, *MMP-3*, *MMP-13*, aggrecanases, and a production of proteoglycans and *Col2a1* in *IL-1β*-stimulated OA chondrocytes treated with celecoxib or GS [16,22,35,36,51,52,53,54,55,56], while no data from the literature are available concerning the combination of celecoxib and GS on cartilage turnover.

Finally, our experience focused on a deeper investigation on the potential mechanism of action underlying favorable effects of celecoxib and GS combination on chondrocytes metabolism through analysis of the NF-κB signaling pathway.

It is known that NF-κB proteins constitute a family of ubiquitously expressed transcription factors triggering the expression of inflammatory mediators and matrix-degrading enzymes involved in detrimental processes occurring during OA [57].

A number of studies showed the protective role of GS on cartilage metabolism by inhibiting activation of the NF-κB signaling pathway, as well as its nuclear translocation, in human OA chondrocytes stimulated with *IL-1β* [33]; this effect was also observed when GS was used at a range of concentration similar to that found at plasma concentration, as established by pharmacokinetics studies [37,40].

Growing evidence suggests that celecoxib exerts a direct effect on cartilage metabolism by modulating pathways independent to *COX-2* activity and PGE2 inhibition, probably through the regulation of NF-kB signaling [15,47,58].

Our data are in line with the current literature demonstrating that the treatment of OA chondrocytes with celecoxib or GS reduced NF-kB signal activation as well as *p50* and *p65* subunits nuclear translocation induced by *IL-1β*; this effect was strongly enhanced when the drugs were tested in combination. We further demonstrated that the use of a specific NF-κB inhibitor, BAY11-7082, reduced the effect of *IL-1β* on inflammation, oxidative stress, apoptosis, and cartilage turnover, potentiating the activity of celecoxib and GS, as previously reported [54]. This allows us to speculate the hypothesis that celecoxib and GS could be effective in the regulation of chondrocyte metabolism through a direct inhibition of NF-κB signaling.

Different findings pointed out that GS seems to exert its effect on NF-κB-dependent transcription via an epigenetic mechanism, regulating the demethylation of specific CpG sites of DNA in the IL1β promoter, responsible for the aberrant expression of *MMPs*, *ADAMTS*, and *IL-1β* in human articular chondrocytes [37,54]. On the other hand, it is currently not completely defined how celecoxib mediates the activity of NF-κB, but it is possible to assume that it follows the PI3K/AKT/IKK/NF-kB pathway regulation, implicated in the regulation of apoptosis and cell proliferation, as demonstrated in different studies on cancer cell lines [59,60].

## 4. Materials and Methods

### 4.1. Sample Collection and Cell Cultures

Human OA articular cartilage was collected from femoral heads of five non-obese (body mass index ranging from 20 to 23 kg/m^2^) and non-diabetic patients (two men and three women, age ranging from 65 to 75) with coxarthrosis according to ACR criteria [61], undergoing hip replacement surgery.

OA grade ranged from moderate to severe, and cartilage showed typical disease changes, as the presence of chondrocyte clusters, fibrillation, and loss of metachromasia (Mankin degree 3–7) [62]. The femoral heads were supplied by the Orthopaedic Surgery, University of Siena, Italy. The use of human articular samples was authorized by the Ethic Committee of Azienda Ospedaliera Universitaria Senese/Siena University Hospital (decision no. 13931/18) and the informed consent of the donor.

After surgery, cartilage fragments were aseptically dissected and processed by an enzymatic digestion, as previously described [63]. For growth and expansion, cells were cultured in Dulbecco’s Modified Eagle Medium (DMEM) (Euroclone, Milan, Italy) with phenol red and 4 mM L-glutamine, supplemented with 10% fetal bovine serum (FBS) (Euroclone, Milan, Italy), 200 U/mL penicillin, and 200 µg/mL streptomycin (P/S) (Sigma-Aldrich, Milan, Italy). The medium was changed twice a week, and the cell morphology was examined daily with an inverted microscope (Olympus IMT-2, Tokyo, Japan). OA primary chondrocytes at the first passage were employed for the experiments [64]. For each single experiment a cell culture from a unique donor was used.

### 4.2. Pharmacological Treatment

Human OA chondrocytes were plated in 6-well dishes at a starting density of 1 × 10^5^ cells/well until 85% confluence. Prescription-grade crystalline GS (Dona^®^) and celecoxib (Celebrex^®^) were supplied by Meda Pharma SpA (Viatris group). The powders of the substances were dissolved in the culture medium in phosphate-buffered saline (PBS) (Euroclone, Milan, Italy), according to the instructions, and directly diluted in the culture medium for the treatment to achieve the final concentrations required.

The cells were cultured in DMEM enriched with 0.5% FBS and 2% P/S, and they were treated for 24 and 48 h with celecoxib and GS, at the concentration of 1.85 µM and 9 µM, respectively, to better reproduce their therapeutic effect in vivo. The treatment was performed in the presence of *IL-1β* (10 n g/mL) (Sigma-Aldrich, Italy), added after 2 h of pre-incubation with the drugs; the experiments were also assessed analyzing the combination of both drugs at 24 and 48 h. Afterwards, the cells were recovered and immediately processed to carry out flow cytometry and quantitative real-time PCR, while the supernatant was collected and stored at −80 °C until ELISA assay.

Some cultures were pre-incubated for 2 h with BAY 11-7082 1 μM (NF-κB inhibitor, IKKα/β, Sigma–Aldrich, Milan, Italy) and then treated for 24 h with celecoxib and GS. Afterwards, the gene expression of the target genes (*COX-2, IL-1β, IL-6, TNF-α, MMP-1, MMP-3, MMP-13, Col2a1, BCL2*, *SOD-2, CAT,* and *NRF2*) was evaluated.

### 4.3. Cell Viability

The viability of the cells after pharmacological treatment was evaluated by MTT (3-[4,4-dimethylthiazol-2-yl]-2,5-diphenyl-tetrazoliumbromide) (Sigma-Aldrich, Milan, Italy) for each experimental condition, as previously described [65]. Timing of drug treatment was selected according to the percentage of surviving cells (Figure S1). The percentage of surviving cells was evaluated as (absorbance of considered sample)/(absorbance of control) × 100. Data were reported as OD units per 10^4^ adherent cells.

### 4.4. RNA Isolation and Quantitative Real-Time PCR

OA chondrocyte were grown and maintained in 6-well dishes at a starting density of 1 × 10^5^ cells/well until they became 85% confluent in DMEM supplemented with 10% FBS, before replacement with 0.5% FBS for the treatment. Then, cells were collected, and total RNA was extracted using TriPure Isolation Reagent (Euroclone, Milan, Italy) according to the manufacturer’s instructions. Five hundred nanograms of RNA of target genes was reverse-transcribed by using the QuantiTect Reverse Transcription kit (Qiagen, Hilden, Germany), according to the manufacturer’s instructions. Target genes were assessed by real-time PCR using the QuantiFast SYBR Green PCR kit (Qiagen, Hilden, Germany). Primers used for PCR reactions are listed in Table S1.

All qPCR reactions were achieved in glass capillaries by a LightCycler 1.0 (Roche Molecular Biochemicals, Mannheim, Germany) with LightCycler Software Version 3.5. The reaction procedure has been described in detail in our previous studies [63].

For the data analysis, the Ct values of each sample and the efficiency of the primer set were calculated by LinReg Software and converted into relative quantities [66,67]. The normalization was performed considering Actin Beta (*ACTB*) as a housekeeping gene for the analyzed target genes [68].

### 4.5. ELISA Assay

After the pharmacological treatment, the supernatant was collected and stored at −80 °C until analysis. PGE2 production, the release of *COX-2*, *IL-1β*, *IL-6*, *TNF-α*, *MMP-1*, *MMP-3*, *MMP-13*, and *Col2a1* were detected using ELISA kits (Boster Biological Technology, CA, USA).

*IL-1β* limit of detection was 250 pg/mL. Inter-assay and intra-assay coefficients of variation were 5.7–8.9%and 4.1–7.3%, respectively.

*IL-6* limit of detection was 300 pg/mL. Inter-assay and intra-assay coefficients of variation were 7.2–8.6% and 6.2–7.4%, respectively.

*TNF-α* limit of detection was 1000 pg/mL. Inter-assay and intra-assay coefficients of variation were 5.4–6.4% and 4.8–7.4%, respectively.

*MMP-1* limit of detection was 10,000 pg/mL. Inter-assay and intra-assay coefficients of variation were 7.6–8.3% and 5.8–6.5%, respectively.

*MMP-3* limit of detection was 10000 pg/mL. Inter-assay and intra-assay coefficients of variation were 6.2–6.9% and 6.4–6.9%, respectively.

*MMP-13* limit of detection was 10,000 pg/mL. Inter-assay and intra-assay coefficients of variation were 7.5–8.1% and 6.4–7.0%, respectively.

*Col2a1* limit of detection was 20 ng/mL. Inter-assay and intra-assay coefficients of variation were <10% and <8%, respectively.

### 4.6. Apoptosis Detection

Apoptotic cells were measured by using the Annexin V-FITC and propidium iodide (PI) (ThermoFisher Scientific, Milan, Italy) kit. OA chondrocyte were seeded in 12-well plates (8 × 10^4^ cells/well) for 24 h in DMEM with 10% FBS, before replacement with 0.5% FBS used for the treatment. The apoptosis assay was performed as previously described [69]. A total of 10,000 events (1 × 10^4^ cells per assay) were measured by the instrument. The results were examined with Cell Quest software (Version 4.0, Becton Dickinson, San Jose, CA, USA). Cells simultaneously stained with Alexa Fluor 488 annexin-V and PI were considered for the evaluation of apoptosis (total apoptosis) [22]. The results were represented as percentage of positive cells to each dye.

### 4.7. Mitochondrial Superoxide Anion (•O_2_^−^) Production

OA chondrocyte were seeded in 12 well-plates (8 × 10^4^ cells/well) for 24 h in DMEM with 10% FCS, before replacement with 0.5% FBS for the treatment. The mitochondrial superoxide anion detection has been performed by MitoSOX Red staining as previously described [69]. A density of 1 × 10^4^ cells per assay (a total of 10,000 events) was measured by flow cytometry, and data were analyzed with CellQuest software (Version 4.0, Becton Dickinson, San Jose, CA, USA). Results were collected as median of fluorescence (AU) and represented the mean of three independent experiments.

### 4.8. Statistical Analysis

Three independent experiments were carried out, and the results were expressed as the mean ± standard deviation (SD) of triplicate values for each experiment. Data normal distribution was evaluated by Shapiro–Wilk, D’Agostino and Pearson, and Kolmogorov–Smirnov tests. Flow cytometry, ELISA, and Western blot results were assessed by ANOVA with Bonferroni post hoc test. Quantitative real-time PCR was evaluated by one-way ANOVA with a Tukey’s post hoc test using 2^−ΔΔCT^ values for each sample. All analyses were performed through the SAS System (SAS Institute Inc., Cary, NC, USA) and GraphPad Prism 6.1. A *p*-value < 0.05 was considered as statistically significant.

## 5. Conclusions

In the present study we confirm the anti-inflammatory and anti-catabolic effects of the therapeutic dose of prescription-grade GS on human OA chondrocyte metabolism.

Furthermore, our results demonstrate the chondroprotective role of celecoxib in OA cells, reinforcing the evidence in favor of using this drug as potential DMOADs. In fact, in vitro studies showed the direct effects of celecoxib on cartilage, bone, and synoviocytes metabolism [15], raising the question of whether it is more than an anti-inflammatory and analgesic drug and, thus, has additional disease modifying effects.

A number of clinical studies reported that the combination of celecoxib and GS effectively modulate immune inflammatory response, oxidative stress damage, and joint pain and function in patients with knee OA [70,71,72]. Our results demonstrate, for the first time, the synergistic effect of celecoxib and GS on human OA chondrocyte metabolism. In particular, this combination treatment exerts a protective role on chondrocytes against the detrimental activities induced by *IL-1β* stimulus, reducing inflammation, apoptosis, oxidative stress, and regulating cartilage turnover, and this activity was effective through direct regulation of NF-κB signaling pathway activation.

Taken together, our in vitro findings suggest that the simultaneous treatment of celecoxib and GS seems to be more effective overall than each single treatment alone, for all the evaluated processes. This result may support the use of a combination therapy for the treatment of OA in clinical practice, since attenuating multiple pathways leading to inflammation and joint destruction can facilitate a safe and effective management of OA.

Our data provide indicative interesting results that deserve to be confirmed with further investigations.

Some limitations need to be mentioned. The same analyses on healthy primary chondrocytes are recommended, to better understand the effectiveness of celecoxib and GS on chondrocyte homeostasis and, in particular, their relevance in OA damage. Then, a deeper examination of the molecular mechanism responsible for the pharmacological effects may contribute to find out the exact role of celecoxib and GS in OA.

## Supplementary Materials

The following are available online at https://www.mdpi.com/article/10.3390/ijms22168980/s1.
