# Supplementary material for: A Combination of Celecoxib and Glucosamine Sulfate Has Anti-Inflammatory and Chondroprotective Effects: Results from an In Vitro Study on Human Osteoarthritic Chondrocytes
Source: Int J Mol Sci. 2021 Aug 20;22(16):8980. doi: 10.3390/ijms22168980 (PMC8396455; doi:10.3390/ijms22168980)
Supplement: Supplementary file 1 [file ijms-22-08980-s001.zip › Figures S1-S3.pdf]

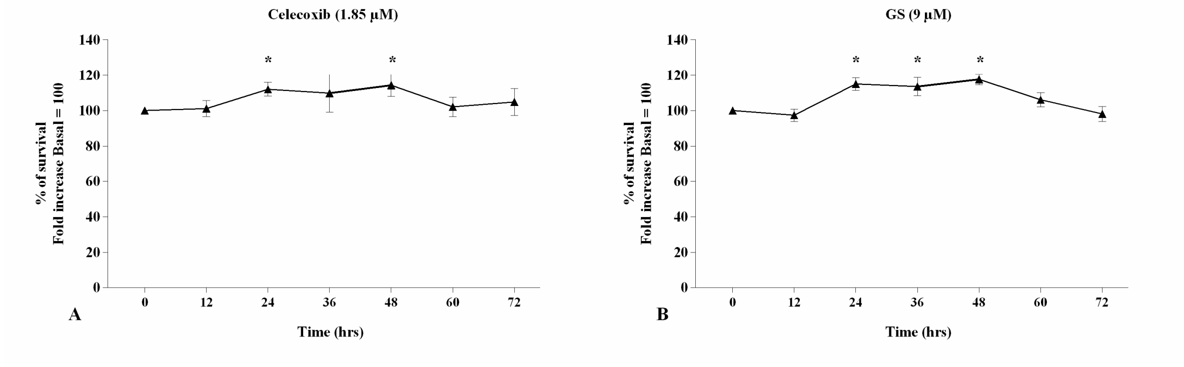

Figure S1. Evaluation of cell viability by MMT assay. Human osteoarthritic (OA) chondrocytes were incubated with celecoxib (1.85 μM) (A) and prescription-grade glucosamine sulfate (GS) (9 μM) (B) for different time points: 0, 12, 24, 36, 48, 60, and 72 h. The percentage of surviving cells was referenced to the ratio of the value of interest and the value of the basal condition, reported equal to 100. Data were represented as mean ± SD of triplicate values. *p < 0.05 versus 0 h.

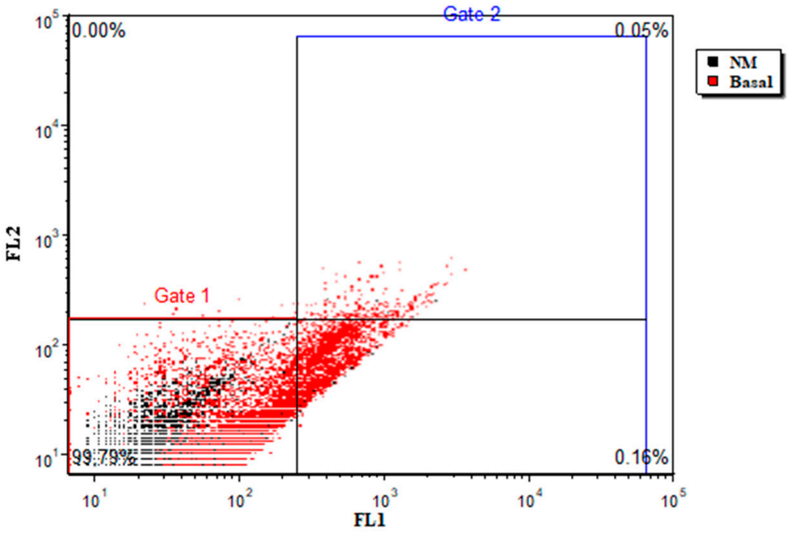

Figure S2. Detection of the apoptosis rate analyzed by flow cytometry and measured with An-nexin V Alexa fluor 488 assay. A representative result showing the percentage of apoptotic, live, and necrotic cells assessed at basal conditions following Annexin V (FL1) and propidium iodide staining (FL2).

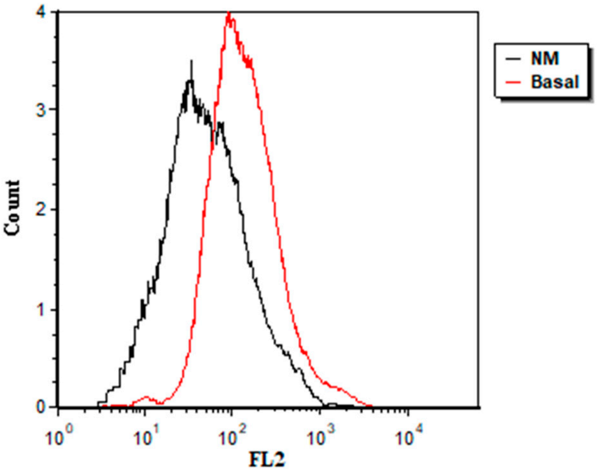

Figure S3. Flow cytometry detection of mitochondrial superoxide anion production with MitoSOX staining at basal conditions (Basal, cells without treatment).
