# Supplementary material for: A Combination of Celecoxib and Glucosamine Sulfate Has Anti-Inflammatory and Chondroprotective Effects: Results from an In Vitro Study on Human Osteoarthritic Chondrocytes
Source: Int J Mol Sci. 2021 Aug 20;22(16):8980. doi: 10.3390/ijms22168980 (PMC8396455; doi:10.3390/ijms22168980)
Supplement: Supplementary file 1 [file ijms-22-08980-s001.zip › Table S1.pdf]

Table S1. List of primers for quantitative real time PCR.

Target Genes	Cat. No. (Qiagen)
COX-2	QT00040586
PGE2	QT00040533
IL-1β	QT00021385
IL-6	QT00083720
TNF-α	QT00029162
BCL2	QT00000721
SOD-2	QT01008693
CAT	QT00079674
NRF2	QT00027384
MMP-1	QT00014581
MMP-3	QT00060025
MMP-13	QT00001764
Col2a1	QT00049518
NFKB1	QT00063791
ACTB	QT00095431
RELA	QT01007370

Abbreviations: *COX-2* = cyclooxygenase, *PGE2* = prostaglandin E2, *IL-1β* = interleukin 1β, *IL-6* = interleukin 6, *TNF-α* = tumor necrosis factor *α*, *BCL2* = B-cell lymphoma 2, *SOD-2* = superoxide dismutase 2, *CAT* = catalase, *NRF2* = nuclear factor erythroid 2 like 2, *MMP-1* = metalloproteinase 1, *MMP-3* = metalloproteinase 3, *MMP-13* = metalloproteinase 13, *Col2a1* = type II collagen, *NFKB1* = Nuclear Factor Kappa B Subunit 1 (p50), *RELA* = Proto-Oncogene, NF-KB Subunit (p65), *ACTB* = Actin Beta.
